# Three cases of esophagorespiratory fistula following neoadjuvant DCF therapy for resectable esophageal cancer

**DOI:** 10.1007/s13691-025-00843-1

**Published:** 2026-01-12

**Authors:** Asuma Ide, Hironori Tsujimoto, Seiichiro Fujishima, Risa Kariya, Naoyuki Uehata, Takafumi Suzuki, Hiroki Tashiro, Keita Kouzu, Yoshihisa Yaguchi, Hideki Ueno

**Affiliations:** Department of Surgery, National Defense Medical College, 3-2 Namiki, Tokorozawa, 359-8513 Japan

**Keywords:** DCF therapy, Esophagorespiratory fistula, Esophageal cancer, Adverse events

## Abstract

Neoadjuvant chemotherapy with 5-FU, cisplatin, and docetaxel (DCF) is standard for resectable esophageal cancer in Japan but is linked to severe adverse events. We report three cases of esophagorespiratory fistulas (ERFs) occurring during DCF therapy. Case 1: A 72-year-old woman (cStage II) developed fever after two DCF cycles. CT and esophagography confirmed an ERF. An esophageal stent was placed, but she later opted for best supportive care. Case 2: A 53-year-old man (cStage IIIB) developed pneumatosis intestinalis and pneumonia nine days into DCF. Imaging confirmed an ERF. He received a stent on day 19 and continued chemotherapy for over a year. Case 3: A 65-year-old man (cStage IIIA) presented with dyspnea eight days after starting DCF. CT revealed pneumothorax and pyothorax. A stent was placed on day 19. His condition initially improved but later declined, leading to best supportive care. DCF therapy may enhance survival but carries significant risk for ERFs. Early recognition of symptoms and rapid intervention are critical to managing complications.

## Introduction

Recent studies, including the Japan Clinical Oncology Group (JCOG1109) trial, have demonstrated that neoadjuvant therapy with 5-fluorouracil (FU), cisplatin, and docetaxel (DCF), followed by esophagectomy, significantly improves overall survival in patients with resectable esophageal cancer compared to the conventional doublet regimen of 5-FU and cisplatin (CF) [1]. DCF therapy has shown a pathological complete response rate of 18.6%, significantly higher than the 2.2% observed with the CF regimen. Therefore, DCF therapy has become the standard preoperative treatment in Japan. However, it is also associated with a high risk of adverse events, particularly hematologic toxicity [2, 3].

A rare but serious life-threatening complication is the development of an esophagorespiratory fistula (ERF), which may result from direct tumor invasion or treatment-induced tissue injury [4]. ERFs occur in approximately 10–22% of patients undergoing chemoradiation therapy [5–8], but reports of ERF arising during chemotherapy without radiation remain limited.

We report three cases of ERF that developed during neoadjuvant DCF therapy in patients with resectable or borderline resectable esophageal cancer.

## Case presentation

### Schedule of DCF therapy

DCF therapy, consisting of 5-FU (750 mg/m^2^/day on days 1–5), cisplatin (70 mg/m^2^ on day 1), and docetaxel (70 mg/m^2^ on day 1), was planned to be administered every 3 weeks for three cycles. Pegfilgrastim was administered on day 3 of each DCF cycle as primary prophylaxis.

### Criteria for continuing chemotherapy after the occurrence of ERF

The criteria for continuing chemotherapy after the occurrence of ERF were the absence of active infection, improvement in performance status following interventions such as stent placement, antibiotic therapy, and drainage, and the patient’s request to resume chemotherapy.

### Case 1

**Fig. 1:**
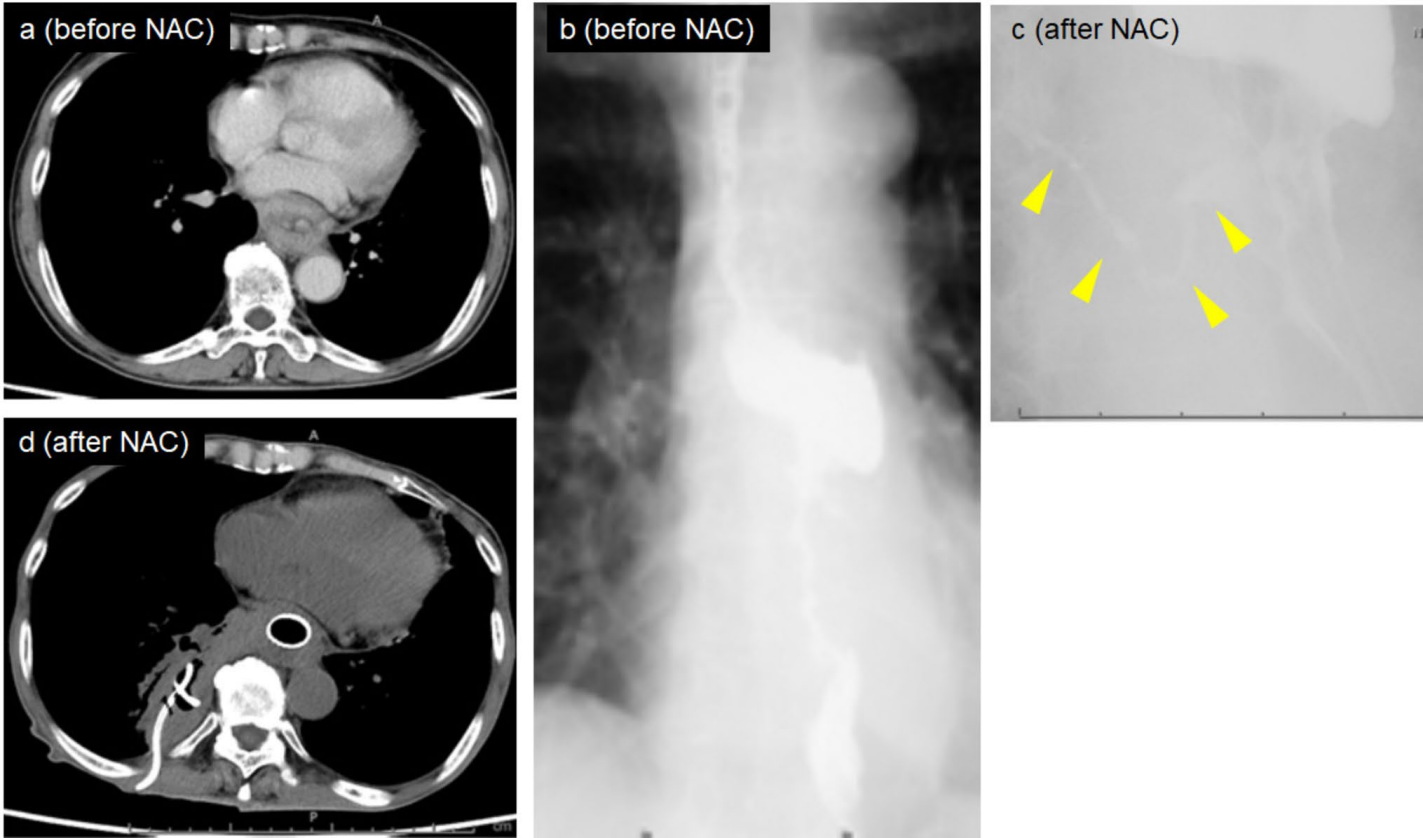
Clinical course images of Case 1. (**a**) Contrast-enhanced CT scan showing a tumor in the middle and lower thoracic esophagus. (**b**) Esophagography prior to DCF showing lower thoracic esophageal stenosis. (**c**) Esophagography after two cycles of DCF therapy showing persistent stenosis and an esophagorespiratory fistula (arrowhead). (**d**) CT image showing esophageal stent and right-sided pyothorax drainage. NAC: neoadjuvant chemotherapy

A 72-year-old woman was diagnosed with esophageal squamous cell carcinoma (SCC) (MtLt cT3r N0 M0 cStage II, according to the Japanese Classification of Esophageal Cancer [9]). Endoscopy before DCF therapy revealed an ulcerative, circumferential lesion extending from the middle to the lower thoracic esophagus with significant luminal narrowing. CT demonstrated esophageal wall thickening measuring more than 9 cm in length, without clear invasion of adjacent organs, and the tumor was considered resectable (T3r). PET-CT showed intense FDG uptake (SUVmax 16.1) with no evidence of distant metastasis. She developed a fever after two cycles of DCF therapy (26 days after initiation). Laboratory data showed a white blood cell (WBC) count of 15,500/μL and C-reactive protein (CRP) of 12.2 mg/dL. CT revealed that the ERF extended into the right lower lobe, and esophagography confirmed the presence of the ERF. An esophageal stent was placed on day three of hospitalization, and the right pyothorax was drained. Despite receiving two additional cycles of chemotherapy with an immune checkpoint inhibitor, the patient ultimately opted for best supportive care. No distant metastases were observed during follow-up. (Fig. 1).

### Case 2

**Fig. 2:**
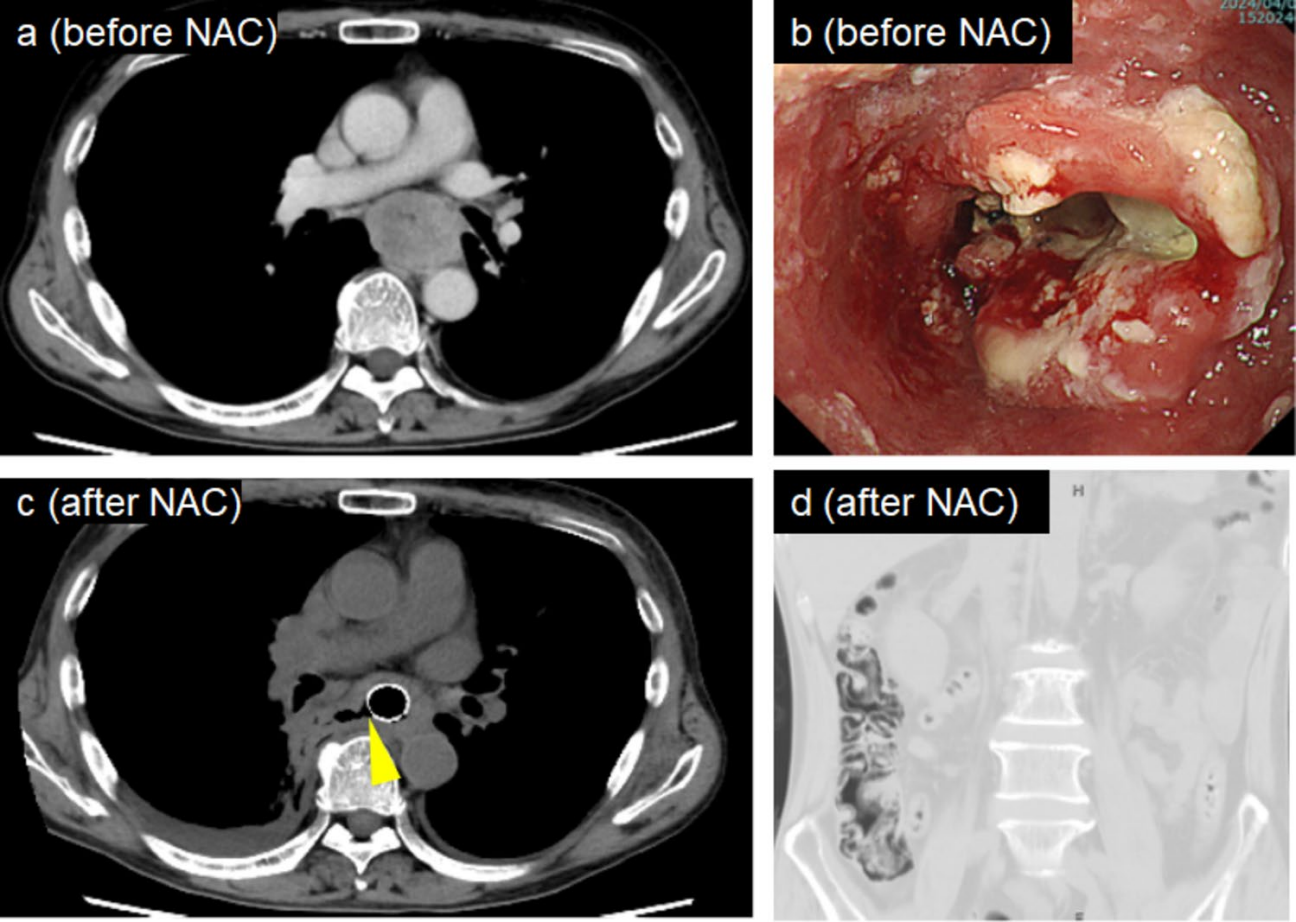
Clinical course images of Case 2. (**a**) Contrast-enhanced CT scan showing a tumor in the middle thoracic esophagus. (**b**) Endoscopic image showing a type 3 tumor in the middle thoracic esophagus. (**c**) CT scan showing right-sided pyothorax and an esophagorespiratory fistula (arrowhead). (**d**) CT scan showing pneumatosis intestinalis at ascending colon. NAC: neoadjuvant chemotherapy

A 53-year-old man was diagnosed with esophageal SCC (Mt cT3br N1 M0 cStage IIIB). Endoscopy revealed an ulcerative lesion on the right side of the lower thoracic esophagus. CT demonstrated esophageal wall thickening exceeding 15 cm in length, abutting the left main bronchus, and the tumor was considered borderline resectable (T3br). PET-CT showed intense FDG uptake (SUVmax 10.3), with no evidence of distant metastasis. He developed pneumonia and pneumatosis intestinalis nine days after initiating DCF therapy. Laboratory data showed WBC count of 9,100/μL and an elevated CRP level of 25.0 mg/dL. CT revealed air adjacent to the esophagus. Esophagography demonstrated contrast leakage into the bronchi, confirming the presence of ERF. An esophageal stent was placed on day 19 after hospitalization. Chemotherapy combined with immune checkpoint inhibitors was continued for over a year; however, pulmonary metastasis developed during the course of treatment, and systemic therapy with paclitaxel was subsequently administered. (Fig. 2).

### Case 3

**Fig. 3:**
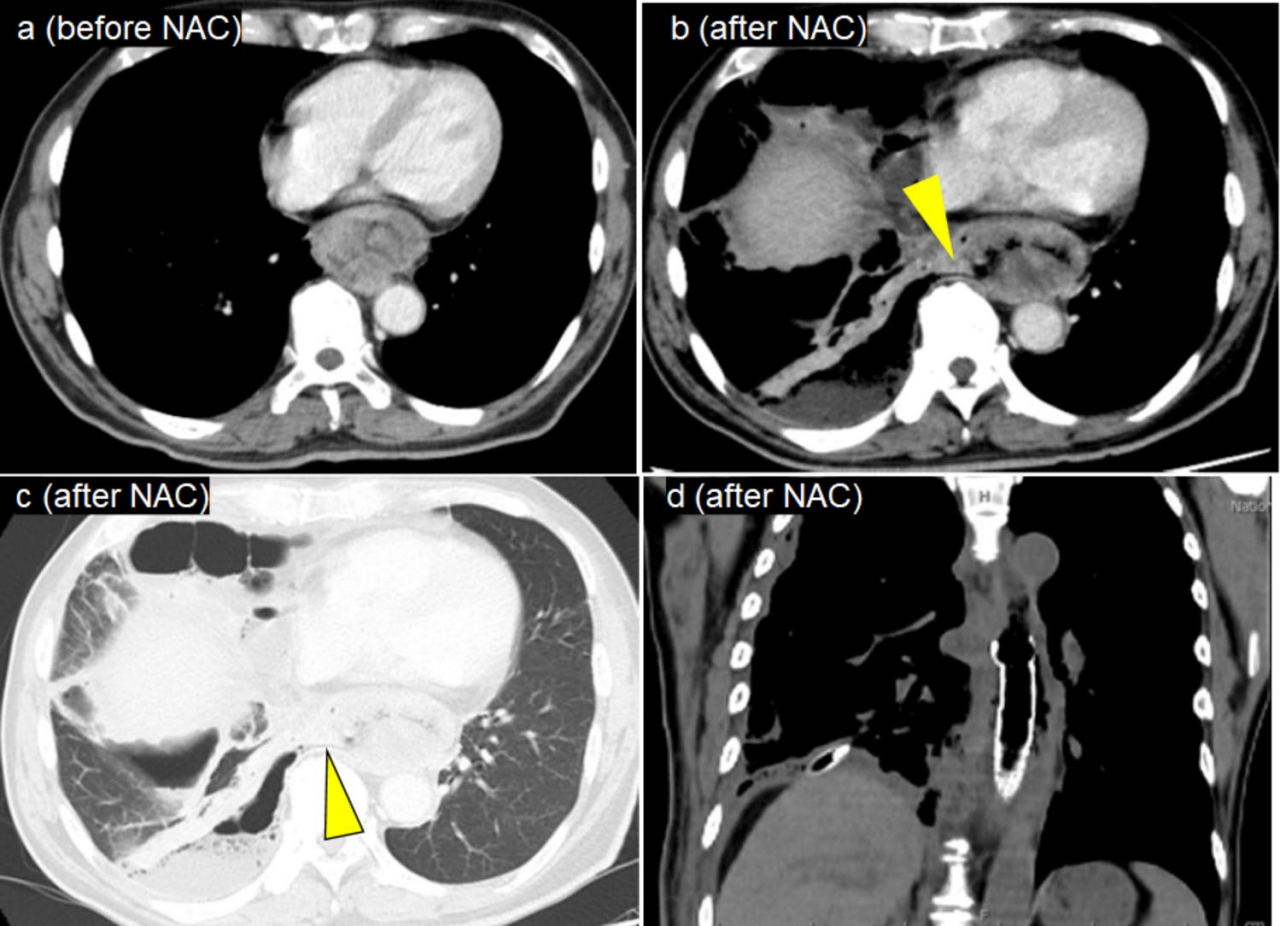
Clinical course images of Case 3. (**a**) Contrast-enhanced CT scan showing a tumor in the middle thoracic esophagus. (**b**, **c**) CT scans showing right-sided pneumothorax, pyothorax, and an esophagorespiratory fistula (arrowhead). (**d**) CT image showing esophageal stent and drainage for right-sided pyothorax. NAC: neoadjuvant chemotherapy

A 65-year-old man was diagnosed with esophageal SCC (Lt cT3r N3 M1a cStage IVA). Baseline endoscopy demonstrated a bulky tumor occupying the lower thoracic esophagus. CT revealed esophageal wall thickening measuring more than 10 cm in length, without apparent invasion of adjacent organs, and the tumor was considered resectable (T3r). PET-CT showed intense FDG uptake in the primary tumor (SUVmax 25.8) and in the left supraclavicular lymph nodes (No. 104R). No additional distant metastases were identified. He developed dyspnea eight days after DCF therapy initiation. Laboratory data showed WBC count of 1,700/μL and an elevated CRP level of 21.4 mg/dL. CT revealed right-sided pneumothorax and pyothorax. An esophageal stent was placed on day 19 after hospitalization, and both pneumothorax and pyothorax were drained. Despite initial improvement, the patient’s performance status declined, and he was transitioned to best supportive care. No distant metastases were observed during follow-up. (Fig. 3).

**Fig. 4:**
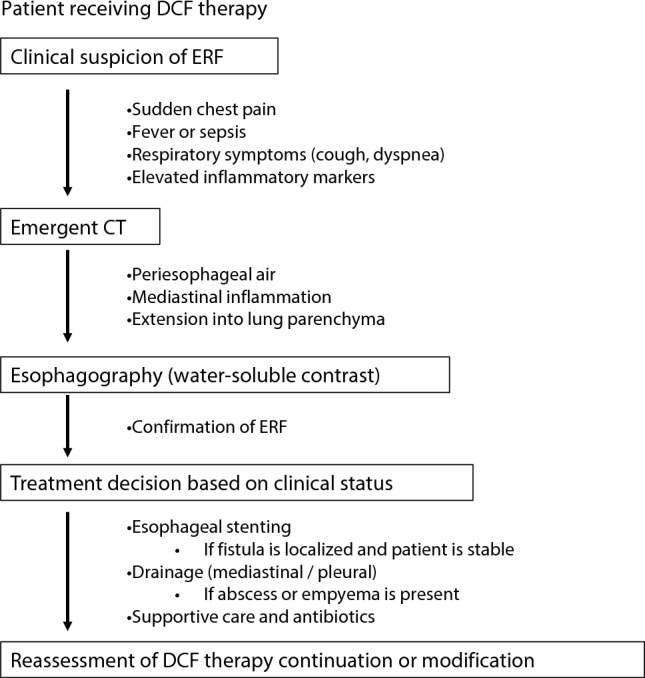
Clinical management flowchart for suspected esophagorespiratory fistula during DCF Therapy. ERF, esophagorespiratory fistula; DCF, 5-fluorouracil + cisplatin + docetaxel

A summary of the clinical findings in the three cases and cases without ERF is presented in Table 1. All cases shared common features, including an bulky tumors and tumor lengths of up to 90 mm. Figure 4 shows the proposed clinical management flowchart for patients with suspected esophagorespiratory fistula during DCF therapy.

**Table 1:** Summary of clinical findings data for the three cases and cases without ERF

	Case 1	Case 2	Case 3	Without ERF (N = 32)
Age	72	53	65	69.5 ± 7.6
Sex	F	M	M	M:F = 27:5
Maim tumor location	Mt	Mt	Lt	Ut/Mt/Lt = 4/20/8
Tumor depth	T3r	T3br	T3r	T1/T2/T3r/T3br = 1/3/16/6
Maximal tumor size (mm)*	38.6	40.7	49.3	34.0 ± 10.7
Tumor length (mm)	90.8	152.5	106.8	60.2 ± 27.6
BMI	15.1	19.9	20.7	20.9 ± 2.7
Albumin (g/dL)	3.3	2.9	3.2	4.0 ± 0.4
Brinkman index	500	0	1600	680.2 ± 657.1
Co-morbidity	No	No	No	Yes/No = 20/12
Cardiovascular	No	No	No	Yes/No = 15/17
Diabates	No	No	No	Yes/No = 4/28
Respiratory	No	No	No	Yes/No = 3/29

ERF, esophagorespiratory fistula

## Discussion

ERF is a life-threatening complication of esophageal cancer treatment [6]. Although well documented in the context of chemoradiotherapy, ERF is rarely reported during chemotherapy without radiotherapy, particularly in association with DCF therapy [10, 11].

In this report, all three patients developed ERF within days to weeks after initiating DCF therapy. Common clinical features included respiratory symptoms, imaging findings of periesophageal air or pleural complications, and fistula confirmation via esophagography. These observations suggest that DCF may precipitate ERF through mechanisms such as rapid tumor shrinkage, and local mucosal inflammation and injury.

Little is known about the incidence of ERF following CF therapy for esophageal cancer [12]. In addition, there were also no case reports of ERF after preoperative DCF therapy. Table 2 summarizes previous reports on ERF following chemoradiation therapy or chemotherapy. Notably, unlike chemoradiation, none of the three studies on preoperative DCF therapy reported ERF as an adverse event, suggesting that such events did not occur during the study periods. Since initiating DCF therapy at our institution in February 2022, we have observed ERF in 3 of 35 cases (8.6%). As shown in Table 1, all three cases involved large tumors exceeding 90 mm in length. In contrast to the controlled conditions of clinical trials, DCF therapy in real-world practice—particularly in patients with large tumors—may be associated with a higher risk of ERF. Yokota et al. reported that grade III of ERF occurred in 4.2% of patients with unresectable disease treated with DCF therapy [13]. In the present study, the incidence of ERF during DCF therapy appeared higher than previously reported. Several mechanisms may explain this observation. First, docetaxel has been associated with mucosal toxicity and ischemic changes, which may increase the fragility of the esophageal wall [14]. Second, the strong antitumor efficacy of DCF could induce rapid tumor regression, particularly in tumor components adjacent to the airway, resulting in localized necrosis and subsequent fistula formation [15]. Previous studies have also demonstrated that DCF achieves higher response rates compared with CF regimens [1], which might enhance this risk. Third, our cases involved relatively large tumors, and tumor size has been identified as a risk factor for ERF, especially when microinvasion or peritumoral inflammation is present [16]. When compared with cases treated with preoperative CF therapy, DCF appears to exert a more intense cytotoxic effect [1], potentially augmenting these mechanisms. Taken together, the combination of higher cytotoxicity, accelerated tumor shrinkage, and greater tumor burden may collectively contribute to the higher incidence of ERF in our series.

**Table 2:** Reports of ERF after preoperative chemotherapy or chemoradiotherapy

Authors	Year	Patients number	Treatment	Chemotherapy regimen	Incidence
Zhang	2018	212	Chemoradiotherapy	TP or PF	22/212 (10.4%)
Pao	2021	129	Chemoradiotherapy	Fluoropyrimidine-based or Taxane-based	18/129 (14.0%)
Kato	2024	601	Chemotherapy	DCF	NR*^1^
Lv	2025	255	Chemotherapy	Immunotherapy + chemotherapy	2/253 (0.78%)
Tamba	2025	86	Chemotherapy	DCF	NR*^2^
Hara	2013	42	Chemotherapy	DCF	NR*^3^

ERF, esophagorespiratory fistula; TP, docetaxel + cisplatin; PF, cisplatin + 5-fluorouracil, DCF; 5-fluorouracil + cisplatin + docetaxel, NR; not referred

^*^1 Observation period:

Median follow-up was 50.7 months (IQR 23.8–70.7) for enrolled patients

Definition and evaluation of adverse events:

Adverse events (AE) were monitored throughout treatment and up to 30 days after the final neoadjuvant dose or before new anticancer therapy. AEs were graded using National Cancer Institute Common Terminology Criteria for Adverse Events (CTCAE) version 4.0. Treatment-related AEs included those considered by investigators to be attributable to administered anticancer agents

^*^2 Observation period:

Median follow-up was 19.2 months (range 5.9–55.5 months)

Definition and evaluation of adverse events:

AEs were assessed according to CTCAE version 5.0. Grade ≥ 3 hematological and non-hematological AEs were specifically recorded (e.g., neutropenia, febrile neutropenia, appetite loss)

^*^3 Observation period:

The manuscript reports outcomes including estimated 2-year progression-free survival and overall survival, indicating a minimum observation of up to approximately two years after therapy

Definition and evaluation of adverse events:

AEs were evaluated weekly during chemotherapy. Grading followed CTCAE version 3.0. Toxicities triggering dose reduction or cessation were predefined, such as grade 4 hematological events, grade ≥ 3 mucosal toxicities, and renal criteria for cisplatin

Several studies have evaluated the anatomical distribution of malignant ERF. Burt et al. reported that the trachea was the most commonly affected site (53%), followed by the left main bronchus (22%) and the right main bronchus (16%), whereas only 6% involved the lung parenchyma [17]. ERF is relatively uncommon but can occur when tumor invasion extends directly into adjacent lung tissue [18]. Taken together, these data suggest that the involvement of the right lower lobe in our three cases represents a less typical but previously documented subtype of ERF.

Prompt esophageal stenting, as performed in all three of our cases, is consistent with current palliative management strategies [7]. However, clinical outcomes varied; one patient remained on active therapy, while the others presented clinical deterioration. This variability highlights the severity of ERF and the challenges of managing it, even with prompt intervention.　The management of malignant ERF is primarily aimed at preventing aspiration and restoring oral intake, and esophageal stent placement is considered the mainstay of treatment in appropriate candidates [17]. The optimal timing of intervention remains controversial; however, emergent stenting is generally recommended when patients present with severe aspiration, respiratory compromise, or rapid clinical deterioration. In Case 1, early stent placement on day 3 was chosen due to rapid onset of aspiration pneumonia and hypoxia, necessitating prompt fistula occlusion. In contrast, Cases 2 and 3 were initially managed with antibiotics and supportive care, and stenting was deferred until day 19 once respiratory status had stabilized, consistent with the principle that patients without acute respiratory failure may benefit from short-term conservative stabilization prior to definitive closure [19].　Our experience suggests that early intervention may be preferable in patients with uncontrolled aspiration or extensive pneumonitis, whereas a delayed approach may be reasonable in clinically stable patients to allow optimization of systemic and nutritional status prior to stenting. Ultimately, the timing should be individualized based on respiratory condition, fistula size, tumor burden, comorbidities, and expected tolerance of stent placement.

As the use of DCF becomes more widespread, physicians should maintain a high index of suspicion for ERF in patients presenting fever, respiratory symptoms, or signs of sepsis. Early imaging and esophagography are essential for diagnosis and for facilitating timely intervention.

## Conclusion

Future studies should focus on identifying the risk factors associated with ERF during DCF therapy and on guiding the selection of alternative treatment strategies for high-risk patients.

## Data Availability

The data used in the current study are available from corresponding author upon reasonable request.

## Abbreviations

DCF: 5-Fluorouracil, cisplatin, and docetaxel
CF: 5-Fluorouracil and cisplatin
5-FU: 5-Fluorouracil
CT: Computed tomography
ERF: Esophagorespiratory fistula
WBC: White blood cell
CRP: C-reactive protein

## Declarations

### Permission to reproduce material from other sources

N/A

### Clinical trial registration

N/A
